# Early Autonomic Dysfunction Following Severe TBI and Impact on Cerebral Hemodynamics: A Narrative Review

**DOI:** 10.3390/jcm15020847

**Published:** 2026-01-20

**Authors:** Kristen Monten, Katrina Hon, Emily Scoville, Tetsu Ohnuma, Monica S. Vavilala, Joseph B. Miller, Vijay Krishnamoorthy

**Affiliations:** 1 School of Medicine, Duke University, Durham, NC 27710, USA; 2 Department of Anesthesiology, Duke University, Durham, NC 27710, USA; 3 Department of Anesthesiology and Pain Medicine, University of Washington, Seattle, WA 98109, USA; 4 Department of Emergency Medicine, Henry Ford Health System, Detroit, MI 48202, USA

**Keywords:** traumatic brain injury, autonomic dysfunction, cerebral autoregulation

## Abstract

**Introduction:** Traumatic brain injury (TBI) is a complex condition that may lead to alterations in cerebral hemodynamics. Impairment of cerebral autoregulatory mechanisms, as well as autonomic dysfunction, has been associated with worse patient outcomes after TBI. **Aims:** The purpose of this narrative review is to synthesize current evidence on impaired cerebral autoregulation, autonomic dysfunction, and their relationship with intracranial pressure in TBI. **Findings:** Initial studies examining waveform data have found that impaired cerebral autoregulation and autonomic dysfunction are present in a high proportion of patients after TBI. These are distinct but closely related phenomena, with current evidence suggesting a bidirectional relationship. **Conclusions:** Impaired cerebral autoregulation and autonomic dysfunction are closely associated. The intersection of these mechanisms is a potential target for intervention to improve patient outcomes after TBI. Additional research is needed to further characterize this relationship.

## 1. Introduction

Traumatic brain injury (TBI) is a leading cause of death and disability worldwide. The global incidence of TBI has been estimated at 69 million cases annually [1]. In the US in 2020, the Centers for Disease Control and Prevention (CDC) reported over 200,000 TBI-related hospitalizations and 69,000 deaths [2]. The lifetime prevalence of sustaining a TBI that causes loss of consciousness has been estimated at 18.2%. TBI affects all age groups, with males twice as likely to report a lifetime history compared to females [3]. Globally, falls and road traffic injuries have been identified as principal mechanisms of TBI [1,4].

TBI is a broad category, encapsulating blunt trauma, penetrating trauma, focal injury, diffuse axonal injury, skull fracture, bleeds, and hematomas [5]. Recovery from severe TBI is a time and resource-intensive process. Morbidity and mortality following a TBI may result from primary or secondary injury. Primary injury in TBI refers to the immediate and direct trauma incurred from an external force. Secondary injury refers to the cascade of early autonomic dysfunction, inflammation, metabolic abnormalities, and altered perfusion that can further precipitate neurologic injury [6]. Autonomic dysfunction after TBI has been identified as a key secondary injury mechanism [7], with possible targets for therapeutic intervention [8,9,10,11,12], but further characterization is needed. Current knowledge is limited about the burden of autonomic dysfunction after TBI and its association with impaired cerebral hemodynamics. The purpose of this narrative review is to synthesize our current understanding of autonomic dysfunction and impaired cerebral autoregulation after severe TBI, with particular emphasis on the relationship with intracranial pressure (ICP). Furthermore, this review aims to provide a roadmap for further research to characterize autonomic dysfunction and cerebral hemodynamics after TBI, to ultimately find therapeutic targets to improve outcomes in this population.

## 2. Methods

This study is designed as a narrative review and intends to comment on the current state of research into cerebral autoregulation and autonomic dysfunction after severe TBI. We prioritized studies specifically examining heart rate variability (HRV) metrics and pressure reactivity index (PRx) in a severe TBI population, and, while in alignment with the current state of the literature, did not utilize a systematic methodology. Relevant studies were identified through iterative searches of PubMed and SCOPUS for combinations of terms “paroxysmal sympathetic hyperactivity”, “PSH”, “early autonomic dysfunction”, “autonomic dysfunction”, “autonomic”, “heart rate variability”, “HRV” with “cerebral hemodynamics”, “cerebral perfusion”, “cerebral edema”, “cerebral autoregulation”, “ICP” or “intracranial pressure” and “TBI” or “traumatic brain injury” or “head injury” or “acquired brain injury”. Studies in languages other than English, as well as studies with non-human subjects, were excluded. Initially, several broad searches were conducted to explore the relationship of Paroxysmal Sympathetic Hyperactivity (PSH) with TBI, associations between TBI and cerebral autoregulation, mechanisms of cerebral autoregulation, and measurement strategies of cerebral autoregulation. For these searches with several hundred results, such as “TBI” and “pressure reactivity index,” a representative sample of recent reviews and key early descriptive papers were read and included. Iterative searches for relationships between autonomic dysfunction, TBI, and cerebral hemodynamics using the above terms and various Boolean operators resulted in few studies. Due to the small number of search results, in these cases searches for additional studies by the same authors, as well as citation searching, were also performed.

## 3. Pathophysiology of Autonomic Dysfunction After TBI

### 3.1. Mechanisms of Autonomic Control

The autonomic nervous system (ANS) is the division of the nervous system that regulates involuntary processes such as blood pressure, heart rate, respiration, and temperature. Autonomic dysfunction in TBI can arise from primary or secondary injury to structures associated with the ANS. While the precise anatomical loci of these centers are not fully established, the anterior hypothalamus, medullary centers, and mesencephalon are frequently cited as primary areas that produce sympathoexcitatory tone. Cortical structures, such as the insular cortex and dorsolateral prefrontal cortex, are largely considered to play an inhibitory and regulatory role on these sympathetic loci [13,14]. The following section reviews hypothesized frameworks of autonomic dysfunction following severe TBI. These frameworks are not mutually exclusive; rather, they describe complementary and interactive contributors to autonomic dysfunction.

### 3.2. Hypotheses of Autonomic Dysfunction Following TBI

Disconnection Theory: Disconnection theory asserts that autonomic dysfunction in severe TBI is caused by the disruption of connections between subcortical sympathetic excitatory centers and inhibitory cortical regions. Damage to the cortex results in disconnection between these structures and massive sympathetic activation [13,15]. Consistent with this framework, diffuse axonal injury and deep cortical lesions are associated with a high incidence of autonomic dysfunction [14]. However, this framework assumes intact pathways downstream of the sympathoexcitatory structures, as extensive injury caudal to these centers would attenuate sympathetic outflow [13]. In addition, structural disconnection alone does not explain the episodic and stimulus-triggered nature of syndromes like PSH.

Excitatory–Inhibitory Imbalance: The excitatory–inhibitory ratio (EIR) model expands on the disconnection theory by proposing that loss of structural connections creates a relative imbalance between excitatory and inhibitory signals within the spinal cord and brainstem. In this model, sympathetic overactivity is attributed to an excessively high ratio of excitatory-to-inhibitory input in the spinal cord. Excitatory input generally comes from afferent stimuli, and inhibitory input from nearby spinal cord regions or supraspinal control. A loss of inhibitory control, an increase in excitation, or both, can increase EIR [13,16]. Spinal sensitization is a central idea of the EIR model, in which stimuli generate exaggerated excitatory postsynaptic potentials, reduced activation thresholds, prolonged neuronal firing, and interpretation of innocuous stimuli as noxious [17], culminating in a widespread, disproportionate sympathetic response [8,16]. This model offers an explanation for persistent autonomic dysfunction even after structural disconnections have healed, and the episodic and stimulus-triggered nature of sympathetic overactivation. However, in vivo structural evidence for this model remains limited.

HPA Axis Dysfunction: Dysfunction of the hypothalamic–pituitary–adrenal (HPA) axis is a recognized contributor to autonomic dysfunction in severe TBI, and provides support for the EIR framework [18]. One study demonstrated evidence of disproportionate, phasic activation of the HPA axis in the context of stimulus-triggered sympathetic storms. During paroxysmal episodes, catecholamine levels increased 200–300% with moderate increase in ACTH and cortisol, suggesting that the relationship between HPA activation and sympathetic output becomes dysproportional in these subjects [18]. This is consistent with the EIR model of sympathetic hyperexcitability. However, catecholamine levels are not reflective of overall severity of autonomic dysfunction [19], indicating that although HPA axis hyperreactivity contributes to autonomic paroxysms, it is not the singular driver of autonomic instability.

## 4. Epidemiology of Autonomic Dysfunction After TBI

Autonomic dysfunction after TBI encompasses a broad spectrum of abnormalities in sympathetic and parasympathetic regulation, and remains incompletely characterized. Clinical presentation may vary significantly, making identification difficult. Autonomic dysfunction has been associated with several organ systems, and may present as isolated organ damage, such as stress cardiomyopathy or acute kidney injury, or as widespread, concurrent multi-organ dysfunction [20]. Recent studies report extracranial multi-organ dysfunction in up to 68% of patients after moderate-to-severe TBI [20].

Paroxysmal Sympathetic Hyperactivity (PSH) is one specific, severe clinical phenotype of autonomic dysfunction observed after TBI, marked by episodes of hyperthermia, hypertension, tachycardia, tachypnea, and/or motor posturing often diagnosed between several days and two weeks post-injury [7]. PSH has been found to occur in 8–33% of TBI patients [21]. Although not all patients with TBI develop PSH, upwards of 80% of PSH cases are attributed to TBI. This suggests TBI is the primary, but not only, inciting type of acute brain injury that can result in PSH. Brain tumors, stroke, and anoxic brain injury have also been implicated [22]. Within subtypes of TBI, diffuse axonal injury has been most highly associated with PSH incidence [8]. In 2014, the Paroxysmal Sympathetic Hyperactivity Assessment Measure (PSH-AM) scale introduced diagnostic criteria for PSH [23]; however, diagnosis remains a challenge due to symptomatic overlap with several conditions, including sepsis, seizures, hydrocephalus, neuroleptic malignant syndrome, and serotonin syndrome [21]. Many patients hospitalized for TBI also receive sedation, which can mask or alter autonomic activity [22]. The episodic nature of PSH can also lead to missed cases. These diagnostic challenges likely apply both to the specific clinical sydrome of PSH, as well as other cases of autonomic dysfunction, which presently lack standardized diagnostic criteria. Given these challenges, it is likely the true incidence of autonomic dysfunction after TBI remains unknown.

## 5. Measures of Autonomic Dysfunction

Granular cardiac waveform data offers an opportunity to better identify and describe autonomic dysfunction that may not be clinically evident, or may not meet critera for a PSH diagnosis. Parameters such as heart rate variability (HRV) and baroreceptor sensitivity (BRS) are widely utilized measures of autonomic function [24]. These metrics provide an opportunity to examine beat-to-beat variation in autonomic function. HRV consists of several time and frequency domain parameters, including SDNN (standard deviation of N-N intervals), RMSSD (root mean square of successive intervals), LF/HF ratio (low frequency to high frequency ratio), HF power, and LF power, that each reflect specific aspects of the autonomic nervous system [24]. Generally, decreased HRV indicates autonomic dysfunction [19]. Exploration of these parameters can provide insight into ANS function and dysfunction in TBI.

## 6. Pathophysiology of Impaired Cerebral Autoregulation After TBI

### 6.1. Mechanisms of Cerebral Autoregulation

In normal physiologic states, cerebral autoregulation is an interplay between myogenic, metabolic, and neurogenic regulatory mechanisms that maintain relatively constant cerebral perfusion across a range of systemic mean arterial pressures (MAP) [25,26]. Cerebral perfusion pressure (CPP) refers to the difference between MAP and intracranial pressure (ICP). Myogenic regulation involves pressure-dependent vasoconstriction and vasodilation of smooth muscle cells in the cerebral vasculature. Metabolic regulation refers to changes in cerebral blood flow (CBF) in response to local metabolic needs, such as increased PaCO_2_ levels or increased local neuronal firing. Neurogenic regulation refers to autonomic innervation of cerebral vessels [25]. The relative contribution of each mechanism to human cerebral autoregulation remains incompletely defined. One study employed a pharmacologic blockade of sympathetic, cholinergic, and myogenic mechanisms to assess relative contributions, which together constituted 62% of the pressure–flow relationship [27]. Metabolic regulation and other mechanisms such as endothelial-cell regulation [28] were not assessed, and likely contribute to the unexplained remaining 38% [27].

### 6.2. Hypotheses of Impaired Cerebral Autoregulation Following TBI

It has been proposed that neurogenic autoregulatory mechanisms are secondary to myogenic and metabolic mechanisms in normal physiologic states [25]. In contrast, in pathophysiologic states or times of significant stress, such as TBI, the autonomic nervous system seems to play an important modulatory role. In several studies, stellate ganglion block, a method of sympathetic blockade, has been associated with increased CBF [26]. Pharmacologic sympathetic blockades have also been associated with increased CBF in settings of increased MAP, as well as increased CO_2_ reactivity [26]. These studies support a modulatory role for the sympathetic nervous system in managing cerebral autoregulation in response to physiologic stressors, although a few studies have also demonstrated inconsistent effects of stellate ganglion blockade and pharmacologic sympathetic blockade on CBF [26]. One study also identified sympathetic autoregulation as a key player in maintaining CBF even within normal physiologic conditions [27].

Although impaired autoregulation is well-recognized after TBI [29,30,31,32,33,34], unknowns remain about its underlying molecular mechanisms. Research into genetic polymorphisms associated with worse outcomes after TBI indicates that several mechanisms, including neuroinflammation, blood–brain barrier dysfunction, metabolic dysfunction, cortical spreading depression, and autonomic dysfunction, are likely involved [28].

## 7. Measures of Cerebral Autoregulation

Cerebral autoregulatory status has previously been assessed by a range of methods across studies, including measurement of CBF responses to pharmacologically increased MAP or tilt-testing [29,30,31], as well as derived measures such as mean flow index (Mx) [32] and pressure reactivity index (PRx) [33,34]. For the purposes of this review, we focus on PRx as a widely accepted measure of impaired cerebral autoregulation [35,36]. PRx measures the association between MAP and ICP, with positive values indicating impaired autoregulation [35].

## 8. Epidemiology of Impaired Cerebral Autoregulation After TBI

Reported prevalence of impaired cerebral autoregulation varies widely across pediatric and adult TBI patients [29,30,31,32,33,34]. Recently, severe TBI has been associated with impaired PRx in 92% of patients, increased ICP in as many as 76% of patients, and impaired CPP in 55% of patients [34]. PRx values in TBI may differ across age and sex. In a review of 10 studies, 8 studies found a relationship between increasing PRx and increasing age. Additionally, 3 of 10 studies identified higher PRx values among females compared to males, although further research is needed [37]. PRx values have not been found to be significantly affected by commonly utilized sedative medications or vasopressors [38].

Impaired autoregulation after TBI has been associated with increased mortality in both adult and pediatric populations [33,39,40]. In adults, time spent above a variety of PRx thresholds (>0, >0.25, or >0.35) was independently associated with 6-month mortality [39]. In pediatric patients, elevated PRx was consistently associated with poor outcomes on the Pediatric Glasgow Outcomes Scale (GOS-E), and a PRx threshold of > 0.25 was most predictive of mortality [40]. A large descriptive study of 822 adults with TBI identified increased mortality in patients experiencing concurrent impairments in ICP, CPP, and PRx, as compared to isolated impairments in ICP or CPP [34]. These relationships further indicate an important role for PRx in modulating TBI outcomes. Current research supports a mitigating role for intact cerebral autoregulation, and a precipitating role for impaired cerebral autoregulation, in secondary injury and outcomes following TBI.

## 9. Associations Between Cerebral Autoregulation, Autonomic Dysfunction, and Intracranial Pressure

Both autonomic dysfunction and impaired cerebral autoregulation have been independently associated with increased mortality and worse outcomes in TBI [41,42,43]; however, the intersection between these complex phenomena, and their relationship with ICP, remains incompletely described. Several early studies have sought to begin examining this relationship using autonomic parameters such as HRV and BRS, in association with PRx and ICP.

### 9.1. Early Evidence for a Bidirectional Relationship Between ICP, HRV, and BRS

In patients with refractory intracranial hypertension secondary to TBI or subarachnoid hemorrhage, relationships have been observed between increasing ICP and ANS activation, as measured by rises in BRS and HRV. Specifically, 15 patients were noted to have a causal relationship from ICP to ANS, and 5 from ANS to ICP [44]. These findings suggest a bidirectional relationship between ICP and the ANS. Activation of the ANS in cases of refractory intracranial hypertension supports the previously postulated notion that ANS activation, or neurogenic autoregulation, is a key fallback autoregulatory system especially in times of physiologic stress [26]. It has been hypothesized that the inverse relationship observed in this study, ANS activation resulting in increased ICP, is an example of pathologic ANS activation secondary to worsening acute brain injury.

### 9.2. Evidence for a Relationship Between ICP and PRx

The relationship between ICP, CPP, and cerebral autoregulation is similarly nuanced. In a review of 13 studies, 11 studies identified a significant relationship between PRx and ICP in TBI. Only two studies found no relationship [45]. Several of these studies grouped patients by ICP levels, and found higher PRx values in the elevated ICP groups. Across studies, calculated correlation coefficients between ICP and PRx ranged from 0.11 to 0.44 [45]. Two studies also explored the relationship between ICP signal complexity and PRx, and found an association between impaired signal complexity and increased PRx values. Interestingly, two studies demonstrated uncoupling of the relationship between ICP and PRx values at both very low and very high ICP levels [45]. This finding aligns with our traditional understanding of cerebral autoregulation, in which autoregulatory mechanisms function over a range of systemic blood pressures [25,26]. Severe derangements in ICP reflect overwhelm of these mechanisms.

Another study in this review grouped patients by PRx values, and found patients with very elevated PRx values (>0.3) had very elevated maximum ICP values. Patients with 0.2 > PRx < 0 had the lowest ICP values. Interestingly, patients with PRx values < 0 and >0.3 did not significantly differ in minimum ICP values [45]. This finding suggests cerebral autoregulation plays a role in modulating states of elevated ICP, up to a certain threshold; however, it is still possible to have evidence of autoregulatory impairment without concurrent elevations in ICP. In a study comparing TBI to acute subarachnoid hemorrhage, patients with both injuries had worse outcomes at higher levels of ICP. However, only TBI patients had poorer outcomes when PRx values were >0 [46]. This suggests the relationship between ICP and PRx in TBI may be unique compared to other neurologic insults. Elevated PRx, and thus impaired cerebral autoregulation, is likely a key risk factor for significant secondary injury after TBI, and, in several studies, appears to predispose patients to dangerous elevations in ICP.

### 9.3. Intact Cerebral Autoregulation Is Protective

Normal PRx values appear to have protective effects against secondary pressure injury following TBI. In one study, the number of episodes of elevated ICP > 30 mmHg correlated directly with both duration and number of elevated PRx episodes [47]. In another study examining ICP and PRx in the setting of vasopressor-induced MAP increases, ICP burden, defined as the net change in ICP during 15 min intervals of MAP > 100 mmHg, was lowest in patients with intact PRx (<0.25). ICP burden was also decreased in patients at >3 days post-injury, as compared to patients within the first 3 days of injury, as well as patients who did not have significant hypotension at time of initial injury [48]. It has been proposed that PRx values may best reflect impairment in cerebral autoregulatory systems in the first few days post-injury, although the temporality of this relationship remains incompletely characterized [49]. 

### 9.4. Associations Between HRV, BRS, and PRx

Many studies examining the relationship between autoregulation and autonomic dysfunction are limited by sample size (Table 1) [41,42,44,45,50,51]. Among 18 TBI patients, PRx > 0.2 and low total HRV spectral power were independently associated with increased mortality. Increased PRx and lower maximum HF HRV power were also significantly correlated with one another [41]. HF HRV power is a reflection of parasympathetic drive; this result indicates impaired cerebral autoregulation in patients with impaired parasympathetic tone [41]. This finding was replicated in a recent study as well, in which higher PRx was associated with both lower HF HRV power and a lower BRS in patients with TBI [33]. These findings together support a relationship between impaired autoregulation and autonomic dysfunction; however, efforts to utilize HF HRV to predict increased PRx, or impairments in autoregulation, so far demonstrate only slight predictive power [41]. Similarly, among 47 moderate-to-severe TBI patients who underwent monitoring of autonomic variables and PRx for several days after TBI, variables such as BRS, LF/HF ratio, and vLF HRV were closely related to PRx, but tests for the directionality of these relationships revealed mixed results [50]. Together, these findings support a nuanced relationship between autonomic variables and cerebral autoregulation.

### 9.5. Temporal Relationship Between HRV, BRS, and PRx

The temporality of the relationship between impaired autoregulation and autonomic variables in TBI requires further exploration. Early efforts to characterize this relationship demonstrate that in patients with poor outcomes following TBI, BRS stayed low and PRx values rose during increases in ICP over the first 7 days post-injury. In contrast, in patients with good outcomes, BRS increased or demonstrated a U-shaped pattern over 7 days. Concurrently, ICP remained <22 mmHg and PRx < 0 [51]. Both BRS and PRx were predictive of mortality in the first few days after injury [51]. Plots of lowest BRS and highest PRx values for each patient were averaged across time, and demonstrated that BRS values reached their lowest at 2 days post-injury, and PRx values reached their highest at 2.5 days post-injury [51]. The close proximity of these variables in time further suggests an association between autoregulation and autonomic dysfunction.

### 9.6. Paroxysmal Sympathetic Hyperactivity (PSH) and Impaired Autoregulation

A recent study investigated this relationship by retrospectively differentiating TBI patients by PSH status. Patients with PSH had lower heart rates and higher LF HRV power in their first 5 days of ICU admission [33]. PSH and non-PSH patients did not significantly differ in episodes of increased ICP or decreased CPP. There was also no significant difference in the prevalence of impaired autoregulation, measured by PRx, between the PSH and non-PSH groups [33]. Even so, a relationship between PRx, BRS, and HRV parameters was observed. In cases of impaired PRx (>0.3), BRS and HF HRV power were also low. In contrast, when PRx was preserved (<0.3), higher PRx values were correlated with measures of higher HRV (increased RMSSD and SDNN) [33]. These findings suggest autonomic function and cerebral autoregulation are distinct, though closely related, entities. As evidenced by several studies, the relationship between these phenomena is highly complex, and it is therefore possible that these reciprocal relationships between PRx and measures of HRV reflect a continuum of pathophysiologic states. A state in which PRx is >0.3 (impaired) and BRS and HRV are low may reflect a state of failure of all cerebral autoregulatory mechanisms. A state in which PRx is still preserved, but approaching the threshold of >0.3, with higher BRS and HRV, may reflect active efforts by a functioning ANS to maintain physiologic autoregulation amid stress.

### 9.7. A Role for Multimodal Monitoring

To date, one larger multimodal monitoring study has been conducted in 262 severe TBI patients to examine the complex associations between ICP/CPP, PRx, BRS, and HRV. In alignment with prior studies, PRx, ICP, BRS, were associated with mortality [42]. Increases in relative power of HF HRV were also independently associated with mortality, as well as correlated with increased ICP and decreased CPP. Relative HF HRV power did not correlate with PRx, but relative LF HRV power did [42]. The association between increases in relative HF HRV power, worse ICP, and increased mortality is somewhat unexpected, given that HF HRV power is traditionally considered to indicate parasympathetic tone [42]. The authors speculate that in the case of severe TBI and intense physiologic stress, increases in relative HF HRV power are more indicative of a highly dysregulated autonomic system overall, as opposed to a distinct increase in parasympathetic over sympathetic drive [42].

### 9.8. Mechanistic Overview

**Figure 1:**
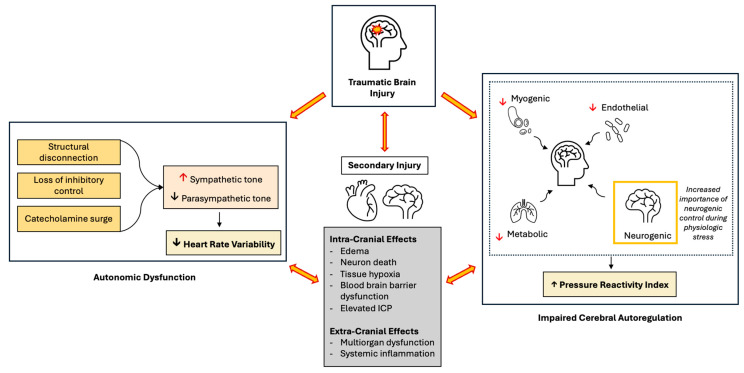
Conceptual model of autonomic dysfunction and impaired cerebral autoregulation following traumatic brain injury. Autonomic dysfunction (left) after TBI may occur due to a combination of structural disconnection between cortical and subcortical structures, excitatory–inhibitory imbalance, and catecholamine surges. Decreased heart rate variability (HRV) quantitatively reflects this dysfunction. Cerebral autoregulation (right) largely relies on coordinated myogenic, endothelial, and metabolic regulatory mechanisms to maintain stable cerebral perfusion. In times of physiologic stress such as severe TBI, however, when myogenic, metabolic and endothelial mechanisms may be overwhelmed (red downward arrows), neurogenic autoregulation has been proposed to play a larger role. Pressure reactivity index (PRx) is a measure of cerebral autoregulatory state, with positive values indicating greater autoregulatory impairment. Although the precise mechanisms linking autonomic dysfunction and impaired autoregulation remain incompletely defined, their close interaction contributes to cerebral hemodynamic instability, elevated intracranial pressure (ICP), and secondary brain injury. In turn, secondary injury may further exacerbate autonomic dysfunction and impaired cerebral autoregulation, perpetuating a harmful cycle.

Together, these studies demonstrate a need for further research to better characterize autonomic dysfunction, cerebral autoregulation, and their effects on intracranial pressure in TBI. Autonomic dysfunction and impaired autoregulation are closely linked mechanisms, as detailed in Figure 1. In TBI, both primary injury and secondary injury processes can disrupt cerebral autoregulation and autonomic control. During periods of severe physiologic stress, such as after TBI, it has been proposed that the autonomic nervous system plays a particularly important role in maintaining hemodynamic stability through neurogenic contributions to autoregulation [25]. However, autonomic dysfunction caused by post-injury catecholamine surges [18] and the loss of inhibitory cortical regulation over brainstem sympathetic centers [16] can impair this important neurogenic mechanism. As a result, cerebral autoregulation can become further compromised. Disturbances in these critical hemodynamic systems can exacerbate secondary brain injury and systemic injury and have been associated with worse patient outcomes [41,42,43].

## 10. Future Directions

Preliminary studies support the presence of a relationship between autonomic dysfunction and impaired cerebral autoregulation in TBI, and a nuanced relationship with ICP. Further research in large patient cohorts is needed to establish the directionality and temporality of this relationship, as well as to improve our ability to identify patients most at risk. A preliminary model to predict the development of PSH utilizing clinical and neuromonitoring data has been described [52]. In the first 3 h post-TBI, increased instability in the correlations between HR and ICP, and between HR and PRx, were associated with the development of PSH [52]. This preliminary study supports a role for early neuromonitoring in predicting patient trajectory. Importantly, this preliminary model was employed specifically to predict PSH, which accounts for only a subset of possible phenotypes of autonomic dysfunction after TBI.

It has been proposed that current guidelines for ICP and CPP thresholds in TBI recovery are insufficient in addressing individual patient needs, especially in patients with evidence of impaired autoregulation [53]. In an initial trial, multimodal monitoring of ICP and ABP in order to calculate personalized optimal CPP targets based on individual patient PRx values has proved safe and feasible [54]. Additional trials are needed to assess the effect of personalized CPP targets on patient outcomes in both the short and long term. In addition to continuous monitoring of PRx, a case can be made for continuous monitoring of HRV metrics. As we describe in this review, HRV metrics have been independently associated with ICP [42,44,51], as well as with PRx values [33,41,42,50,51]. A multimodal monitoring strategy may help identify patients at risk for impaired autoregulation and/or autonomic dysfunction. Early identification may help guide therapy.

Several treatments have shown benefit in addressing autonomic dysfunction after TBI. Temperature management, fluids, vasopressors, and antihypertensives are widely utilized treatment modalities for autonomic dysfunction [9], although further research into vasopressor management is needed [55]. Sedation options such as dexmedetomidine have also been proposed as feasible and safe [10,11]. Some evidence supports a role for baclofen, clonidine, gabapentin, opioids, and propranolol in management as well, though more exploration is needed [9]. Recent evidence also suggests a potential role for stellate ganglion block in mitigating autonomic dysfunction after TBI [12]. While further validation is needed, these interventions aimed at managing autonomic dysfunction may hold promise for improving the trajectory of post-TBI recovery and mitigating secondary injury mechanisms.

## 11. Conclusions

Autonomic dysfunction and impaired cerebral autoregulation are two distinct but closely related phenomena that can contribute to secondary injury after TBI. The relationship between these phenomena appears to be complex and bidirectional. Further studies are needed to explore the time course and directionality of this relationship and its associations with ICP. Multimodal monitoring and interventions aimed at improving autonomic function may have potential to improve patient outcomes.

## Figures and Tables

**Table 1:** Characteristics of studies examining relationship between autonomic variables, cerebral autoregulation, and intracranial pressure after TBI.

Study	Study Aims	*n*	GCS	Parameters	Findings
Lavinio et al. 2008 [41]	Explore relationship between autonomic failure and cerebrovascular reactivity impairment following severe TBI.	18	<8	PRx, frequency domains of HRV (low frequency (LF) spectral power, high frequency (HF) spectral power, total power, LF/HF ratio)	PRx > 0.2 and low spectral power of HRV were associated with fatal outcomes. PRx and HRV spectral power were significantly correlated. HF HRV was mildly predictive of impaired PRx (>0.2).
Sykora et al. 2016 [42]	Test whether autonomic markers are independently associated with functional outcome and mortality. Evaluate the relationships between autonomic function, ICP, and cerebral autoregulation.	262	Median 6	PRx, time domain HRV (SD, SDSD *, RMSSD), frequency domain HRV (LF spectral power, HF spectral power, LF/HF ratio), BRS	Lower BRS and higher relative HF HRV power were independently associated with increased mortality. BRS did not correlate with ICP or CPP, but correlated with PRx in patients > 60 years. Relative HF power of HRV correlated positively with ICP and CPP, not PRx. Relative LF power of HRV correlated significantly with PRx.
Fedriga et al. 2021 [44]	Assess changes in autonomic activity during refractory intracranial hypertension. Explore the temporal relationship between rising ICP and autonomic function.	24 **	Not reported	ICP, time domain (SD, SDSD, RMSSD) of HRV, frequency domain (HF power, LF power, LF/HF ratio) of HRV, BRS	As ICP rose, HRV and BRS increased significantly, with a higher significance level in the HF band. After an “upper breakpoint,” HRV and BRS decreased significantly. In 15 patients, Granger causality tests suggested a directional relationship from ICP to ANS. In 5 patients, the inverse relationship was observed. No significant relationship was observed in 4 patients.
Froese et al. 2022 [50]	Examine the temporal and causal relationship between cerebrovascular reactivity and autonomic function in moderate-to-severe TBI using time-series statistical methods.	47	6	PRx, BPV *** (LF, HF, total spectral power, SD of mean BP, SD of systolic BP, SD of diastolic BP), BRS, HRV (HF, LF, vLF **** spectral power, LF/HF ratio)	Granger causality tests showed bidirectional relationships between PRx and autonomic response. Impulse-response analysis showed that BRS, LF/HF ratio, and vLF HRV had strong temporal associations with PRx. Hierarchical clustering indicated BRS and LF/HF ratio were most consistently associated with PRx.
Uryga et al. 2023 [51]	Investigate individual temporal patterns of BRS over the first 7 days after TBI, and assess how those patterns relate to ICP, PRx, and prognosis.	29	7	PRx, BRS	BRS reached a minimum ~2 days after injury. In patients with good outcomes, BRS significantly increased over 7 days with a “U-shaped” or upward trend. A BRS value ≤ 1.8 ms/mmHg at ~1.5 days post-injury predicted mortality. In patients with poor outcomes, ICP and PRx increased while BRS stayed low.
Burzyńska et al. 2025 [33]	Analyze ANS metrics and cerebral autoregulation in TBI patients with PSH syndrome and assess their prognostic value.	66	6	PRx, BRS, frequency domain HRV (LF power, HF power, total power, LF/HF power), time domain HRV (SDNN, RMSSD)	Cerebral autoregulation was impaired in 67% of patients with PSH vs. 72% without PSH. PSH patients had significantly higher LF HRV and lower HR than non-PSH patients. HR and LF HRV were moderate predictors of PSH. Moderate correlation between impaired PRx and lower BRS, and impaired PRx and lower HF HRV. In intact PRx, increasing PRx values were associated with increased HRV SDNN and increased HRV RMSSD.

* Standard deviation of the difference between sequential beats (SDSD). ** This study assessed acute brain injury; 21 patients had TBI, 3 had subarachnoid hemorrhage (SAH). All had refractory intracranial hypertension (RIH). *** Blood pressure variability (BPV). **** Very low frequency spectral power (vLF).

## Data Availability

No new data were created or analyzed in this study. Data sharing is not applicable to this article.

## Abbreviations

The following abbreviations are used in this manuscript:

TBI	traumatic brain injury
ANS	autonomic nervous system
EIR	excitatory–inhibitory ratio
HPA	hypothalamic–pituitary–adrenal
PSH	paroxysmal sympathetic hyperactivity
MAP	mean arterial pressure
CPP	cerebral perfusion pressure
ICP	intracranial pressure
CBF	cerebral blood flow
Mx	mean flow index
PRx	pressure reactivity index
HRV	heart rate variability
BRS	baroreceptor sensitivity
BPV	blood pressure variability
SDNN	standard deviation of N-N intervals
RMSSD	root mean square of successive intervals
LF	low frequency
HF	high frequency
SDSD	standard deviation of the difference between sequential beats
SAH	subarachnoid hemorrhage
